# IL-10 revisited in systemic lupus erythematosus

**DOI:** 10.3389/fimmu.2022.970906

**Published:** 2022-08-01

**Authors:** Swayanka Biswas, Katja Bieber, Rudolf Armin Manz

**Affiliations:** ^1^ Institute for Systemic Inflammation Research, University of Lübeck, Lübeck, Germany; ^2^ Lübeck Institute of Experimental Dermatology (LIED), University of Lübeck, Lübeck, Germany

**Keywords:** IL-10, B cells, systemic lupus erythematosus, extrafollicular T cells, autoimmunity

## Abstract

IL-10 is a cytokine with pleiotropic functions, particularly known for its suppressive effects on various immune cells. Consequently, it can limit the pathogenesis of inflammatory diseases, such as multiple sclerosis (MS), inflammatory bowel disease, Crohn’s disease, and Epidermolysis bullosa acquisita, among others. Recent evidence however indicates that it plays dual roles in Systemic lupus Erythematosus (SLE) where it may inhibit pro-inflammatory effector functions but seems to be also a main driver of the extrafollicular antibody response, outside of germinal centers (GC). In line, IL-10 promotes direct differentiation of activated B cells into plasma cells rather than stimulating a GC response. IL-10 is produced by B cells, myeloid cells, and certain T cell subsets, including extrafollicular T helper cells, which are phenotypically distinct from follicular helper T cells that are relevant for GC formation. In SLE patients and murine lupus models extrafollicular T helper cells have been reported to support ongoing extrafollicular formation of autoreactive plasma cells, despite the presence of GCs. Here, we discuss the role of IL-10 as driver of B cell responses, its impact on B cell proliferation, class switch, and plasma cells.

## Introduction

In the context of T-dependent (TD) immune responses (i.e. response to protein antigens), B cells can form short-lived plasma cells, memory B cells and long-lived plasma cells, eventually yielding a complex humoral response consisting of antibodies of various immunoglobulin (Ig) subtypes. The antigen-binding affinities of secreted antibodies range from relatively low dissociation constants (Kd) of below 10^−6^ moles/liter to very high Kd values of approximately 10^-10^ moles/liter (1–4). Antibodies against proteins of infectious agents tend to be of highest affinities, whereas autoantibodies can reach affinities of reported Kd of 10^-7^ moles/liter (5).

Low affinity antibodies are formed early in the GC reaction and during the extrafollicular pathway. In the latter, activated B cells directly differentiate into plasmablasts within extrafollicular foci of secondary lymphoid organs, avoiding GC formation (6–8). High affinity antibodies are typically formed in the context of the follicular pathway. There activated B cells initiate a GC reaction transforming a primary follicle into a secondary follicle (GC), eventually yielding hypermutated and affinity selected memory B cells and plasma cells producing antibodies with high affinity. Antibodies formed within both the follicular and the extrafollicular pathway play critical roles in the immediate immune defense against pathogens, as well as the development of autoimmune and allergic diseases (9–14). The extrafollicular antibody response is fast, peaking within a few days after immunization/infection to provide the first line adaptive humoral response. This early response consists mainly of IgM, but can also include a considerable proportion of switched isotypes, such as IgG1 (15). The follicular response generates memory B cells and long-lived plasma cells which can produce antibodies of all subclasses, which could persists for decades (9–14). If the same antigen-activated B cell uses asymmetric division to initiate a follicular and extrafollicular response simultaneously, or if different B cells are attracted into the two pathways is unknown so far (12).

## Sources and functions of IL-10

Cytokines are important co-stimulatory factors that can modulate the nature of TD follicular and extrafollicular responses. They serve as B cell differentiation factors, support the survival of antibody-secreting plasmablasts and plasma cells and are key factors controlling antibody class switch. IL-10 is a major B cell stimulating cytokine, recently reported to be a crucial driver of the extrafollicular B cell response. It is a pleiotropic cytokine with potent immune tolerogenic effects (16). The receptor for IL-10 is a transmembrane spanning molecule comprising of the two subunits IL-10R1 and IL-10R2 (17). After activation and receptor dimerization, the JAK-STAT pathway gets activated resulting in the upregulation of STAT3.

When it was first discovered, IL-10 was described as Cytokine Synthesis Inhibitory Factor (CSIF) because it suppressed the activation and subsequent cytokine production by T helper (Th)1 cells. Originally described as part of the Th2 cytokine pattern, it was later realized that IL-10 is also produced by late Th1 cells (18–20). As of today, the cellular source of IL-10 has been broadened to include many hematopoietic and non-hematopoietic cell types, including B cells, malignant and non-malignant plasmablasts and plasma cells, cytotoxic T cells, natural killer cells, regulatory T cells, dendritic cells, among others (21–26).

One function of IL-10 is to suppress excessive pro-inflammatory action that might result in tissue damage. Accordingly, IL-10 deficient mice develop severe colitis (27). The cytokine inhibits the upregulation of MHC II and co-stimulatory molecules on antigen presenting cells (APC), thereby inhibiting the production of inflammatory cytokines (28, 29). Dendritic cell specific deletion of the IL-10 receptor limits chemokine production in tissues (30). It further prevents unwarranted T cell activation and subsequent proliferation that might result in inflammatory effector functions (31, 32). Plasma cell derived IL-10 has a local impact on adjacent neutrophils and myeloid cells (33, 34). IL-10 production from both B and T cells has important immunosuppressive functions, but while CD4 specific IL-10 KO mice show indications of severe inflammatory bowel disease with a high penetrance (35), there is no such indication in B specific IL-10 KO mice. Hence, indicating that IL-10 production of the two distinct lymphocyte types exhibit non-redundant functions.

IL-10 plays a protective role in MS and in the murine model of this autoimmune disorder of the central nervous system, experimental autoimmune encephalomyelitis (EAE) (36, 37). The cytokine is expressed at increased levels in remission phases of and its murine model EAE (38). B lineage derived IL-10 was shown to be critical for the disease course of EAE, with plasmablasts the critical source of the inhibitory cytokine (31).

In a mouse model for the autoimmune skin blistering disease Epidermolysis bullosa acquisita (EBA), IL-10 blockade has been shown to largely downregulated disease pathogenicity by inhibiting innate effector functions, while induction of IL-10+ plasma cells could inhibit the disease (33). In conclusion, IL-10 is produced by various cell types, has multiple functions, could down-regulate inflammatory effector cells but can also drive inflammation *via* promotion of the production of autoantibodies.

## IL-10 influence of B cell proliferation, differentiation, and Ig class switch

Several cytokines are important co-stimulatory factors for the B cell response (39). Among them is IL-10, which has been known for long to promote B cell activation, differentiation and antibody class switch *in vitro* (40). Recent studies now imply that IL-10 could be of crucial relevance for B cell differentiation in SLE and murine lupus, particularly for the extrafollicular response (41–44).

In an *in vitro* setup using CD40 stimulated human B cells, IL-10 induced B cell proliferation, comparable with the effect of IL-4, which is a cytokine that plays a prominent role for the survival and proliferation of cultures GC B cells and *in vitro* generated GC-like cells (45–47). IL-10 but not IL-4 could stimulate the secretion of antibodies into the culture (45).

A separate study focused on the role of IL-10 on GC B cells, also in an *in vitro* setup. Tonsillar B cells were cultured in the presence of CD40L and a follicular dendritic cell (FDC) like cell line, along with different cytokines. In the presence of IL-10, GC B cells serially differentiated into CD20+ CD38- memory B cells and subsequently into CD20-CD38+ plasma cells. However, in the absence of IL-10, plasma cell differentiation was impaired (48). In another study, cultured tonsillar human B cells, either activated through their Ig receptor or CD40, were seen to undergo proliferation and subsequent differentiation to antibody secreting cells in an IL-10 dependent manner (45). IL-10 can also promote the secretion of IgM, IgG1 and IgG3 in cultures of stimulated naïve tonsillar surface IgD+ B cells (49). A follow-up study than showed that IL-10 initiated the formation of switch circles, i.e. fragments of IgH DNA excised during class-switch recombination, indicating that IL-10 is actually supporting class switch recombination (50). Other studies using cultures of human B cells from peripheral blood or isolated tonsils provided further evidence that IL-10 can support the proliferation, Ig class switch and antibody secretion (51, 52). There is some evidence that B cell derived IL-10 could promote plasma cell differentiation and the secretion of antibodies of the IgM and IgG classes in an autocrine manner (53).

## IL-10+ extrafollicular T helper cells as drivers of a persistent extrafollicular response in SLE

In the GC, B cells interact with a specialized subset of CD4+ T cells called follicular helper T (Tfh) cells which can phenotypically be characterized as CD4+ CXCR5+ ICOS+ PD1+ cells (54). Downregulation of chemokine receptor CCR7 and upregulation of CXCR5 allow Tfh cells to migrate towards B cells of secondary lymphoid tissues in the B cell follicles (55). Here, they interact with the antigen-activated B cells. Tfh cells provide IL-21, a cytokine required for a normal GC function (56). As shown in SLE, IL-10 is of crucial importance to drive the extrafollicular plasma cell formation outside the GC, independent of IL-21 (42). Multiple lines of evidence indicate that the extrafollicular plasma cell response is supported by a T helper cell population distinct from Tfh cells. These extrafollicular T helper cells express PD-1, but lack the expression of the chemokine receptor CXCR5, indicating that they are not able to migrate to the B cell follicles. Instead they express CCR6, with or without expression of CXCR3. Extrafollicular helper T cells do not produce IL-21, but seem to support extrafollicular plasma cell responses and antibody production mainly through IL-10 (42). In SLE patients, extrafollicular helper T cells have been identified in the peripheral blood, in the lymph nodes and tubular interstitial spaces of the kidneys (42, 44). Extrafollicular helper T cells were seen to stimulate plasmablast formation and IgG-autoantibody production of Toll-like receptor stimulated naïve and memory B cells from SLE patients in an IL-10 dependent manner. Moreover, murine IL-10+ extrafollicular helper T cells were shown to provide B cell help and support the production of IgG antibodies in pristane-induced lupus and after immunization with the model antigen ovalbumin, *in vivo*. In these systems, autoantibodies and anti-ovalbumin-antibodies were approximately 30% and 50% reduced in B cell specific IL-10 receptor knock out mice, respectively. Hence, indicating that B cell help from extrafollicular helper T cells is indeed partly dependent on IL-10 (42). Similarly, T cell derived IL-10 seems to support the extrafollicular antibody response to infection with plasmodium (57).

## The opposing roles of IL-10 in SLE

There have been numerous studies indicating that IL-10 may play multiple and opposing roles in murine lupus. As discussed above, there is good evidence that IL-10 supports the proliferation and differentiation of autoreactive B cells into plasma cells in SLE, thereby contributing to disease progression (42, 44).

However, studies in murine models indicate that IL-10 can also play a protective role in lupus. Research done on lupus-prone B6.Sle1.Sle2.Sle3 mice depicted elevated levels of IL-10 from B lineage cells and CD4+ T cells. Transduced continuous overexpression of low levels of IL-10 in skeletal muscle cells resulted in a delay of autoantibodies. It further resulted in reduced renal pathology as depicted by reduced amounts of IgG and C3 deposits in the glomeruli (58). Because increased IL-10 impaired the activation of T cells in this model, it is difficult to draw conclusions on the direct role of IL-10 on B cell function. However, it indicates that the anti-inflammatory role of IL-10 plays a beneficial role in the disease. Accordingly, B cell specific deletion of IL-10 in Lyn deficient mice, another lupus model, lead to increased disease severity, but did not alter plasma cell counts (59). Hence indicating that B cell derived IL-10 can inhibit lupus pathology but has no impact on the overall B cell activation. In B6.NZMc1c4 mice, yet another lupus model, genetic deletion of IL-10 lead to increased production of autoantibodies (60). If this was due to a direct effect IL-10 has on B cells, or the consequence of the well-known inhibition of T helper cell differentiation (56), remains to be established.

The dual and opposing roles IL-10 has in lupus was recently depicted in New Zealand Black x New Zeeland White F1 mice were *in vitro* experiments revealed pro- and anti-inflammatory IL-10 effects (61). *In vivo* blockade of IL-10 starting after start of the disease at the age of 5 months increase production of autoantibodies and lupus pathogenesis (61). In another study however, continuous administration of blocking anti-IL-10 antibodies at birth, delayed the onset of autoantibody production and also disease symptoms such as proteinuria, glomerulonephritis (62). Together, these studies indicate that IL-10 blockade had opposing effects on the development of autoantibodies when applied before disease onset or afterwards. Since the extrafollicular response is expected to precede the follicular response, it is possible that early blockade of IL-10 blocks the initiation of the pathogenic extrafollicular B cell response hence delaying the onset of the disease. Blockade of IL10 later on may still inhibit the extrafollicular response, but the IL-10 independent follicular response could take over. IL-10 inhibition may even boost the follicular response because of the inhibitory effect the cytokine has on the formation of CD4 T helper cells. Hence, it is possible that after disease onset the IL-10 blockade shifts the balance between the extrafollicular and the follicular response, without changing the overall production of autoantibodies too much.

## Conclusion

**Figure 1:**
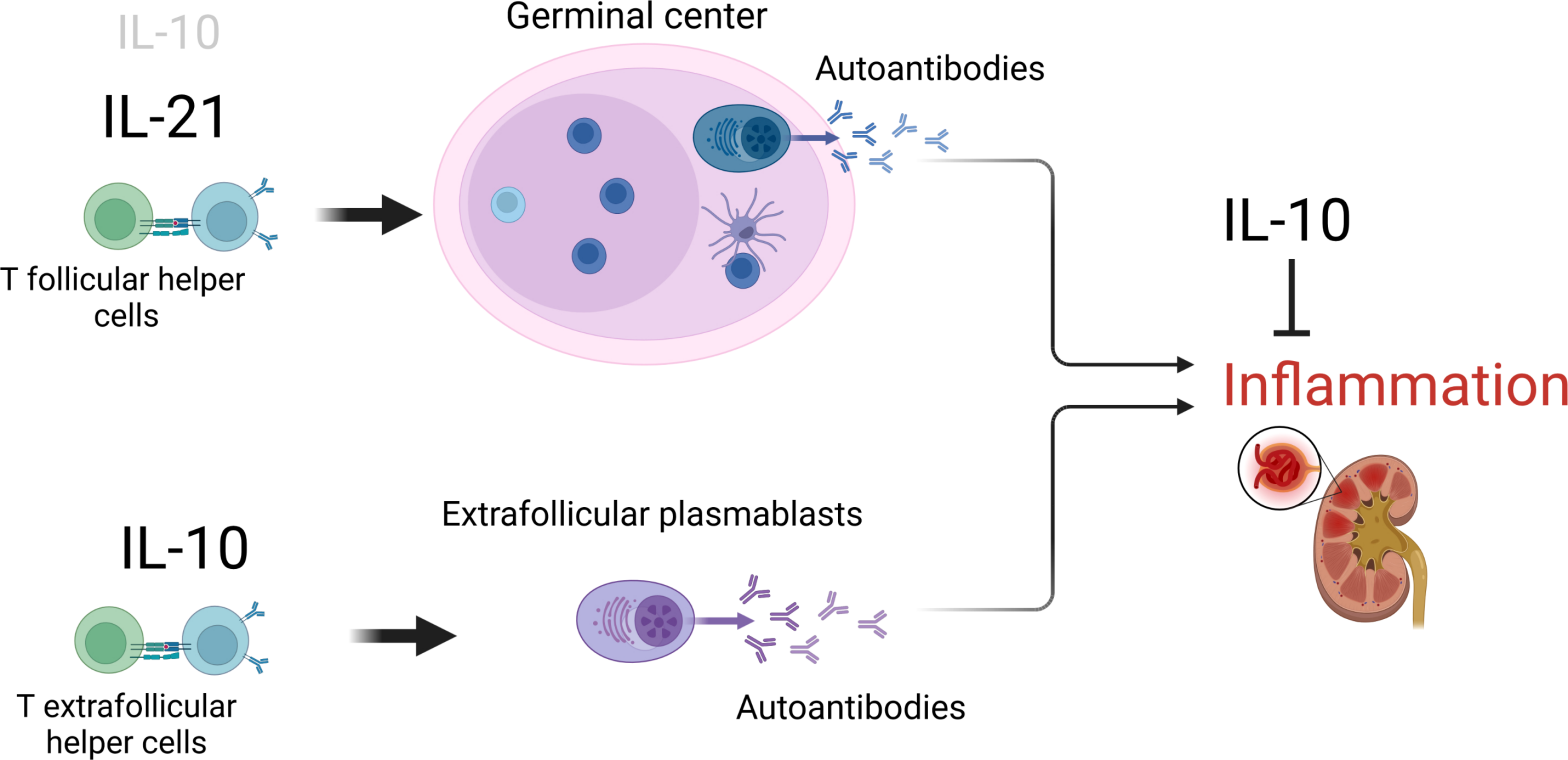
The opposing role of IL-10 in SLE. The cytokine drives the continuous formation of pro-inflammatory autoantibodies, particularly outside GCs, but can also limit inflammatory effector functions. Figure created with BioRender.com.

IL-10 is a potent suppressor of inflammatory effector functions. However, like several other cytokines, it can also promote B cell proliferation, differentiation and class switch. It had been recognized for long that IL-10 plays important, but redundant functions in the follicular B response in GCs. Recent evidence indicates that the cytokine is of even greater importance as a promoter of the extrafollicular B cell response outside the GC (**Figure 1**). In SLE, a specialized subset of extrafollicular helper T cells seems to be crucial to drive the extrafollicular autoantibody B cell response and disease pathogenesis, *via* IL-10. If that holds true also for other autoimmune diseases, remains to be elucidated.

## Author contributions

RM and SB performed the literature search and drafted the original manuscript. SB drew the figure. KB revised the manuscript. All the authors approved the final version of the manuscript.

## Funding

This study was supported by the Deutsche Forschungsgemeinschaft, through the research training group RTG 2633 “Autoimmune Pre-Disease” project A02.

## Conflict of interest

The authors declare that the research was conducted in the absence of any commercial or financial relationships that could be construed as a potential conflict of interest.

## Publisher’s note

All claims expressed in this article are solely those of the authors and do not necessarily represent those of their affiliated organizations, or those of the publisher, the editors and the reviewers. Any product that may be evaluated in this article, or claim that may be made by its manufacturer, is not guaranteed or endorsed by the publisher.
